# Thrombotic Thrombocytopenic Purpura (TTP)–Like Illness Associated with Intravenous Opana ER Abuse — Tennessee, 2012

**Published:** 2013-01-11

**Authors:** Ellyn Marder, David Kirschke, Donna Robbins, John Dunn, Timothy F. Jones, Judy Racoosin, Leonard Paulozzi, Art Chang

**Affiliations:** Tennessee Dept of Health; Div of Anesthesia, Analgesia, and Addiction Products, Center for Drug Evaluation and Research, Food and Drug Administration; Div of Unintentional Injury Prevention, National Center for Injury Prevention and Control; Div of Environmental Hazards and Health Effects, National Center for Environmental Health, CDC

On August 13, 2012, a nephrologist reported to the Tennessee Department of Health (TDH) three cases of unexplained thrombotic thrombocytopenic purpura (TTP), a rare but serious blood disorder characterized by microangiopathic hemolytic anemia and thrombocytopenia. The annual incidence is approximately 1 per 100,000 population (1,2). Known risk factors for TTP include infection with Shiga toxin–producing *Escherichia coli* (STEC) and the use of drugs, including platelet aggregation inhibitors, quinine, and cocaine (1,3,4). The three patients were intravenous (IV) drug users who resided in a rural county in northeast Tennessee. To identify other cases of TTP-like illness that might be associated with injection-drug use, TDH conducted a statewide investigation. By the end of October, a total of 15 such cases had been reported; none were fatal. A case-control study was conducted, and investigators determined that the cases of TTP-like illness were associated with dissolving and injecting tablets of Opana ER (Endo Pharmaceuticals), a recently reformulated extended-release form of oxymorphone (an opioid pain reliever) intended for oral administration. Fourteen of the 15 patients reported injecting reformulated Opana ER. Seven of the 15 were treated for sepsis in addition to TTP-like illness. Twelve patients reported chronic hepatitis C or had positive test results for anti-HCV antibody. Health-care providers who prescribe Opana ER and pharmacists who dispense it should inform patients of the risks from the drug when used other than as prescribed. Health-care providers should ask patients with TTP-like illness of unknown etiology about any IV drug abuse. Suspected cases can be reported to public health officials.

## Clinical Characteristics

Following report of the initial three cases, TDH contacted infectious disease specialists, dialysis centers, and the regional poison center in Tennessee seeking additional cases. A case of TTP-like illness was defined as microangiopathic hemolytic anemia (hemolytic anemia based on haptoglobin and lactate dehydrogenase and the presence of schistocytes) and thrombocytopenia in a person with a hospital admission platelet count ≤50,000/*μ*L, in the absence of certain known causes of TTP. By the end of October 2012, a total of 15 cases had been reported in Tennessee. TDH interviewed patients in person and reviewed medical charts. Among the 15 patients, 13 were women. All were white; none were pregnant. The 15 patients ranged in age from 22 to 49 years (median: 34 years). The earliest diagnosis of TTP-like illness was April 16, 2012 (Figure). Seven of the 15 patients were from the same rural county in northeast Tennessee; five were from nearby counties, and three were from counties in middle Tennessee.

The 15 patients were further categorized by presence or absence of a concurrent infection (as evidenced by sepsis) as a possible etiology. Clinical characteristics were similar among patients with and without infection (Table). Patients reported symptoms typical of TTP-like illness, including nausea (11 patients) abdominal pain (11), fatigue (10), and fever (six). Seven patients were treated for sepsis. Twelve were treated with plasmapheresis. The median admission platelet counts for patients without and with infection were 20,000/*μ*L (range: 9,000–40,000/*μ*L) and 26,000/*μ*L (range: 9,000–49,000/*μ*L), respectively. Activity levels of the von Willebrand factor–cleaving protease (ADAMTS13), which is involved in blood clotting, were available for eight of the 15 patients. ADAMTS13 median activity level among patients without infection was 90% (range: 84%–131%) and among patients with infection was 64% (range: 42%–100%). Twelve of the 15 patients reported chronic hepatitis C or had positive results for anti-HCV antibody testing performed during hospital admission (Table). None were HIV-positive. TDH conducted serologic testing on six patients to assess exposure to STEC O157; one had evidence of prior infection.

## Case-Control Study

To test for an association between TTP-like illness and injection of reformulated Opana ER, TDH conducted a case-control study. Controls were recruited from patients in a methadone clinic in eastern Tennessee and had to meet the inclusion criterion of injection-drug abuse in the previous 6 months. Only drug abuse reported during TDH interviews was used in the analysis. All 15 case-patients and 28 controls participated in the case-control study. No case-patients or controls eligible for the study refused to participate.

Among the 28 controls, median age was 31 years (range: 19–52 years); 13 were female, and all were white. None of the controls received a diagnosis of TTP–like illness. Nine reported injecting Opana ER in the preceding 6 months, including seven who reported injecting reformulated Opana ER. One control was unsure of the formulation and was categorized in the analysis as injecting reformulated Opana ER. Therefore, eight of the 28 controls, compared with 14 of the 15 case-patients, reported recent injection of reformulated Opana ER (odds ratio [OR] = 35.0; 95% confidence interval [CI] = 3.9–312.1). Thirteen of the 14 case-patients who reported reformulated Opana ER abuse injected intravenously; one reported subcutaneous injection. Case-patients reported a first injection of reformulated Opana ER 21–120 days before hospital admission (median: 60 days). The last reported injection of reformulated Opana ER occurred 0–2 days before admission (median: 1 day). None of the case-patients reported using quinine, either alone or as part of the preparation process. Five case-patients also reported IV abuse of hydromorphone or oxycodone; one reported cocaine use. Twenty-two of the 28 controls reported injecting oxycodone, and 18 reported injecting morphine.

Seven of the eight case-patients without infection (as evidenced by sepsis) compared with eight of the 28 controls reported recent injection of reformulated Opana ER (OR = 17.5; CI = 1.8–166.0). One case-patient without infection did not report Opana ER use during the TDH interview but did report use to health-care providers. The odds ratio for case-patients with infection was undefined because all seven with infection reported recent injection of reformulated Opana ER.

## Public Health Response

TDH submitted an alert via CDC’s Epidemic Information Exchange (Epi-X) on August 23, 2012. The Food and Drug Administration (FDA) released a statement regarding the association of IV abuse of reformulated Opana ER and TTP-like illness on October 11. TDH submitted a second alert to Epi-X on October 24, and CDC released a Health Advisory on October 26 to warn against injection of Opana ER and to aid in case finding.

### Editorial Note

TTP is a one of the thrombotic microangiopathies, conditions characterized by thrombosis in arterioles and capillaries that manifest clinically with thrombocytopenia and microangiopathic hemolytic anemia (3). Patients with TTP require hospitalization and usually plasmapheresis. Without treatment, TTP is associated with a high mortality rate (2). TTP is more common among women (1,2). In addition to platelet aggregation inhibitors, other toxic chemotherapeutic and immunosuppressive drugs have been associated with TTP (1,3).

Hepatitis C and systemic infections often are associated with IV drug abuse as well as with thrombocytopenia, hemolytic anemia, and deficiency of the ADAMTS13 enzyme. Therefore, whether TTP was caused by infection or some noninfectious exposure has been unclear in certain previous cases (5,6). However, in the cases described in this report, injection of reformulated Opana ER was strongly associated (OR = 35.0; CI = 3.9–312.1) with the illness of the case-patients.

FDA approved Opana ER for oral use in 2006. However, like other opioid analgesics, the drug has been abused by some persons seeking its euphoria-inducing effects, including some who have crushed the tablets to snort them or dissolved them for injection (7,8). The new formulation, designed to inhibit crushing and dissolving tablets, was released into the market in February 2012. The new formulation contains inactive ingredients not found in the original formulation, including polyethylene oxide (PEO) and polyethylene glycol. Of note, in October 2010, the makers of OxyContin, another extended-release opioid analgesic, also launched a reformulated product designed to deter abuse that contained PEO. No cases of TTP-like illness following injection of reformulated OxyContin have been reported.

It is unclear what component or components of reformulated Opana ER might trigger TTP-like illness when injected and whether different methods of preparing the drug can increase or decrease the risk from injection. No human studies have evaluated the risk from injecting this new formulation, although in one study in rats, intravenously injected PEO caused thrombocytopenia (9). It is also possible that the pills in the Tennessee cases were adulterated by a drug dealer. However, at least two patients obtained the drugs directly from a licensed pharmacy with prescriptions, and the involved communities are far enough apart to make a single nonmedical source for other cases unlikely. In general, injection of any opioid pain reliever formulated for oral use presents risks for fatal toxicity and bloodborne infection, but this is the first report of TTP-like illness associated with abuse of an opioid pain reliever by injection.

What is already known on this topic?

Thrombotic thrombocytopenic purpura (TTP) is a rare but serious blood disorder characterized by microangiopathic hemolytic anemia and thrombocytopenia and has not been associated previously with intravenous abuse of Opana ER, an extended-release form of oxymorphone intended for oral administration. In February 2012, a new formulation of Opana ER was released with the intent to inhibit crushing and dissolving the tablets.

What is added by this report?

In 2012, 15 cases of TTP-like illness were identified among intravenous drug users in Tennessee, including 14 who reported injecting reformulated Opana ER. A case-control analysis identified a strong association (odds ratio = 35.0; 95% confidence interval = 3.9–312.1) between TTP-like illness and injection of reformulated Opana ER.

What are the implications for public health practice?

The disease mechanism and extent of the problem with Opana ER abuse are unknown. Health-care providers should ask patients with TTP-like illness of unknown etiology about injection-drug abuse. Additionally, health-care providers who prescribe Opana ER and pharmacists who dispense it should inform persons using it of the risks involved when used other than as prescribed.

FDA has warned* that Opana ER is meant to be taken orally and should only be taken when prescribed and as directed. CDC has recommended† that clinicians treating patients with TTP-like illness with unknown etiology ask about IV drug abuse, perform a urine drug test to look for oxymorphone, and request a copy of the patient’s prescriptions for controlled substances from state prescription drug monitoring programs. Clinicians should counsel patients who report injection of reformulated Opana ER of the risk for recurrent TTP, bloodborne infections, and overdose with continued use; refer them to substance abuse treatment programs, and notify other clinicians who have prescribed the patient Opana ER. Cases can be reported to state or local health departments. A standardized case report form is available at e-mail, lbp4@cdc.gov.

## Figures and Tables

**FIGURE:**
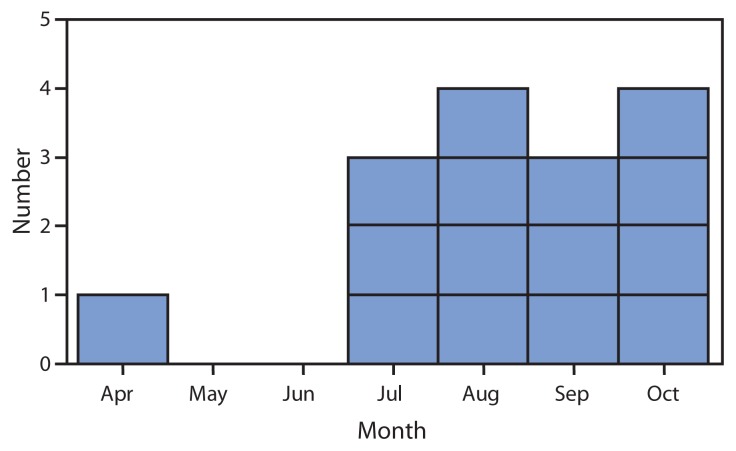
Number of cases (N = 15) of thrombotic thrombocytopenic purpura (TTP)–like illness, by month of first presentation — Tennessee, 2012

**TABLE:** Clinical characteristics of patients with thrombotic thrombocytopenic purpura (TTP)–like illness, by infection status (as evidenced by sepsis) — Tennessee, 2012

	TTP-like illness without infection (n = 8)		TTP-like illness with infection (n = 7)	
		
Characteristic	Median	Range	Median	Range
**Test result**				
Platelet count (per *μ*L)	20,000	9,000–40,000	26,000	9,000–49,000
Hematocrit (%)	18.7	15.3–20.7	19.3	16.6–20.1
Hemoglobin (g/dL)	6.0	5.2–7.3	6.5	5.5–6.7
Creatinine (mg/dL)	1.1	0.5–11.4	2.5	1.2–4.2
BUN (mg/dL)	28	8–84	52	21–59
LDH (units/L)	1,080	131–3,007	768.5	395–1,191
ADAMTS13 activity level (%)	90*	84–131	64*	42–100
Schistocytes present (no. patients)	8	—	7	—
	**No.**	**(%)**	**No.**	**(%)**

**Symptom**				
Nausea	6	75	5	71
Abdominal pain	5	63	6	86
Fever	1	13	5	71
Fatigue	5	63	5	71
**Treatment**				
Plasmapheresis	6	75	6	86
Dialysis	2	25	0	0
**Other illness**				
Hepatitis C	5	63	7	100
Sepsis	0	0	7	100
Endocarditis	0	0	3	43
Renal failure	4	50	7	100

**Abbreviations:** BUN = blood urea nitrogen; LDH = lactate dehydrogenase; ADAMTS13 = the von Willebrand factor–cleaving protease.

* Data not available for seven of the 15 cases.

## Footnotes

* Available at http://www.fda.gov/drugs/drugsafety/ucm322432.htm.

† Available at http://www.bt.cdc.gov/han/han00331.asp.
